# Hemophagocytic Lymphohistiocytosis in the Setting of Progressive Chronic Lymphocytic Leukemia With Five Concurrent Life-Threatening Infections

**DOI:** 10.7759/cureus.83209

**Published:** 2025-04-29

**Authors:** Heenaben Patel, John Patresan, Victoria Krzywicki, Andres Ramirez Gamero, Gerald Colvin

**Affiliations:** 1 Internal Medicine, Roger Williams Medical Center, Providence, USA; 2 Hematology and Medical Oncology, Roger Williams Medical Center, Providence, USA

**Keywords:** chronic lymphocytic leukemia (cll), hemophagocytic lymphohistiocytosis (hlh), infection, invasive aspergillosis, malignancy

## Abstract

Untreated hemophagocytic lymphohistiocytosis (HLH) can be fatal and requires a high degree of clinical suspicion in the setting of an underlying autoimmune disorder, infection, or malignancy. In the presence of multiple infections, it can be challenging to treat the disease, given organ failure and limited options. We present a case of HLH in a patient with chronic lymphocytic leukemia, who was found to have multiple bacterial and fungal infections simultaneously, due in part to construction work on his 200-year-old home.

## Introduction

Hemophagocytic lymphohistiocytosis (HLH) is a disorder of uncontrolled immune activation leading to multiorgan failure [1]. HLH is a rare disease, and it is usually fatal if it remains untreated. Initial diagnosis and further workup for HLH requires a high clinical suspicion, often guided by the available laboratory data. Primary or familial HLH occurs due to genetic defects affecting natural killer cells and cytotoxic T-cells and has autosomal recessive inheritance [2]. Secondary HLH is characterized by unopposed, dysregulated immune system activation in adults secondary to infection, malignancy, or autoimmune disorders [1]. Malignancy-associated HLH cases are mostly seen with lymphoid malignancies, such as B or T cell lymphoma. Very few cases of HLH in patients with untreated long-standing chronic lymphocytic leukemia (CLL) have been reported, which were triggered by disease progression or infection [3-6].

We present a case of HLH seen in untreated CLL, which was triggered by multiple infections with CLL progression. This patient was working on his 200-year-old home and was removing walls, and had significant exposure to molds. He never wore a mask during construction. He presented to our hospital from a community hospital with acute renal failure and respiratory failure. Bronchial alveolar lavage confirmed invasive *Aspergillus fumigatus*, *Candida albicans*, *Pneumocystis jirovecii* (PJP), methicillin-resistant *Staphylococcus aureus* (MRSA), and *Stenotrophomonas maltophilia* pneumonia. He was also found to have *Candida albicans* fungemia. The diagnosis was initially suspected given a very high ferritin level in settings of bicytopenia in critically ill patients [7]. Bone marrow hemophagocytosis is considered a sensitive indicator and may not always be present in all HLH patients at initial evaluation. Although our patient met HLH 2004 criteria for diagnosis initially, bone marrow biopsy results provided additional supportive evidence and favored the HLH diagnosis. CLL is known to cause hypogammaglobulinemia, which can predispose the patient to multiple infections, one of which may have triggered HLH. The rare occurrence of five concurrent infections also makes this case unique in the setting of CLL, given the clinical ambiguity of a possible cause of HLH. The distinction between a driver of HLH, whether it is a malignancy itself or infectious etiology, is challenging to uncover.

## Case presentation

**Figure 1:**
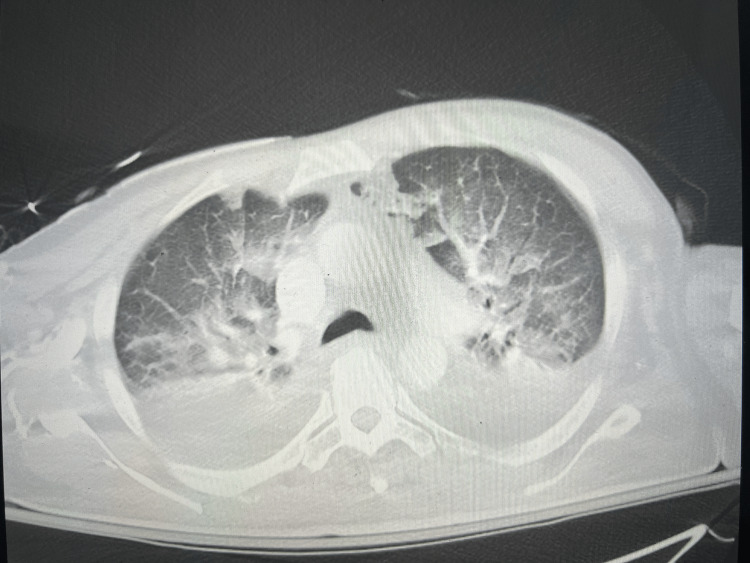
CT angiography pulmonary embolism study showed bilateral confluent airspace disease concerning for infection and moderate-sized pleural effusions bilaterally. No evidence of pulmonary embolism was noted.

A 65-year-old male with a past medical history significant for recently diagnosed Rai stage 0 CLL was transferred from an outside hospital for further management of acute renal failure, respiratory failure, and tumor lysis syndrome (TLS) treatment (Figure 1). He developed generalized weakness, malaise, and fatigue for a few days prior to his first hospitalization. There was no reported fever, chills, night sweats, weight loss, or cough prior to admission. He was found to have massive splenomegaly, 24 cm maximum dimension, and mediastinal and retroperitoneal lymphadenopathy. Initial labs showed white cell count of 91,600/microliter, which progressively decreased to 700/microliter within few days, thrombocytopenia of 33,000/microliter, hemoglobin at 8.4 g/dl, prolymphocyte at 11%, atypical lymphocyte at 94%, uric acid at 28.6 mg/dl, lactate dehydrogenase at 1180 U/L, ferritin at 5372 ng/ml, fibrinogen at 273 mg/dl, which decreased to 95 mg/dl in dive days, creatinine at 4.4, and triglyceride at 294 mg/dl, which increased to 1141 mg/dl (Table 1). Nephrology was immediately consulted upon hospitalization, and the patient was started on hemodialysis. Additionally, bone marrow biopsy was obtained given a history of CLL and a potential concern of transformation to high-grade lymphoma.

**Table 1:** Initial laboratory values during the hospital admission.

Parameters	Result value	Reference range
Hematology		
Total leukocyte count (per µl)	91,600	4000-11,000
Neutrophil (%)	1	40-75
Other - atypical lymphocyte (%)	94	0-0
Hemoglobin (g/dl)	8.4	14-18
Mean cell volume (fl)	97.6	75-100
Platelet count (per µl)	33,000	150,000-450,000
Chemistry		
Creatinine (mg/dl)	4.4	0.6-1.2
Blood urea nitrogen (mg/dl)	146	6.0-20.0
Potassium (mmol/liter)	5.5	3.5-4.9
Calcium (mg/dl)	7.6	8.5-10.5
Anion gap	28	3.0-12.0
Phosphorus (mg/dl)	21.2	2.5-4.6
Albumin (g/dl)	2.9	3.2-5.5
Total bilirubin (mg/dl)	0.7	0.2-1.0
Direct bilirubin (mg/dl)	0.2	0.0-0.2
Indirect bilirubin (mg/dl)	0.5	0.2-0.8
Aspartate aminotransferase (U/liter)	144	10.0-42.0
Alanine aminotransferase (U/liter)	45	10.0-60.0
Alkaline phosphatase (U/liter)	118	42-121

He was initially treated with rasburicase and supportive measures for his TLS, given the lab findings. In the first week, he continued to get hemodialysis while inpatient as per nephrology recommendations. Additionally, he developed neutropenic fever. He was started on broad-spectrum antibiotics. The immunoglobulin (Ig) panel was also checked at this time and revealed IgG level of 1337 mg/dl, IgM level of 29 mg/dl, and IgA level of 204 mg/dl (Table 2). Within a week of hospitalization, he was intubated emergently for acute hypoxic respiratory failure. Bronchoscopy was performed, which revealed negative cytology, but grew *Aspergillus fumigatus*, *Candida albicans*, *Pneumocystis jirovecii*, MRSA, and *Stenotrophomonas maltophilia*.

**Table 2:** Other significant initial laboratory values during hospital admission.

Parameters	Result value	Reference range
Coagulation panel		
Prothrombin time (seconds)	10.9	9.7-11.6
Prothrombin time international ratio	1.02	0.93-1.10
Activated partial thromboplastin time (seconds)	29.1	22.2-33.9
D-dimer (mg/Liter FEU)	34.74	0.19-0.52
Fibrinogen (mg/dl)	273	200-400
Fibrin degradation products	>=20	<5
Hemolytic labs		
Haptoglobin (mg/dl)	100	36-195
Indirect bilirubin (mg/dl)	0.5	0.2-0.8
Reticulocyte (%)	0.48	0.5-2.17
Lactate dehydrogenase total (U/L)	1180	140-271
Uric acid (mg/dl)	28.6	2.6-7.2
Iron studies		
Iron level (µg/dl)	127	50-170
Transferrin (mg/dl)	171	192-382
Ferritin (ng/ml)	5372	23.9-336.2
Other		
Triglyceride (mg/dl)	294	<150
IgG (mg/dl)	1337	635-1741
IgA (mg/dl)	204	66-436
IgM (mg/dl)	29	43-279
Free kappa light chains (mg/L)	122.3	3.3-19.4
Free lambda light chains (mg/L)	63.2	5.7-26.3
Free kappa/lambda ratio	1.94	0.26-1.65
Fungitell (pg/ml)	>500	<80
IL-2R alpha (CD25), soluble (pg/ml)	31,200	532-1891

A lumbar puncture was done and confirmed central nervous system involvement of CLL by flow cytometry, although bacterial, viral, and fungal testing of the cerebrospinal fluid (CSF) was negative. Candidemia was suspected to be secondary to central line-associated bloodstream infection (CLABSI), hence, central lines and dialysis catheters were removed, and the patient completed two weeks of intravenous micafungin 100 mg/day. His transthoracic echo was negative for endocarditis. Patient’s bronchoalveolar lavage (BAL) detected PJP in low numbers (265 copies/mL) and improved without treatment for PJP, so likely this represents colonization. He was already on atovaquone prophylaxis. His BAL showed very high galactomannan and fungitell, suggestive of invasive aspergillosis of the lung, and he received voriconazole 200 mg twice daily for that.

**Figure 2:**
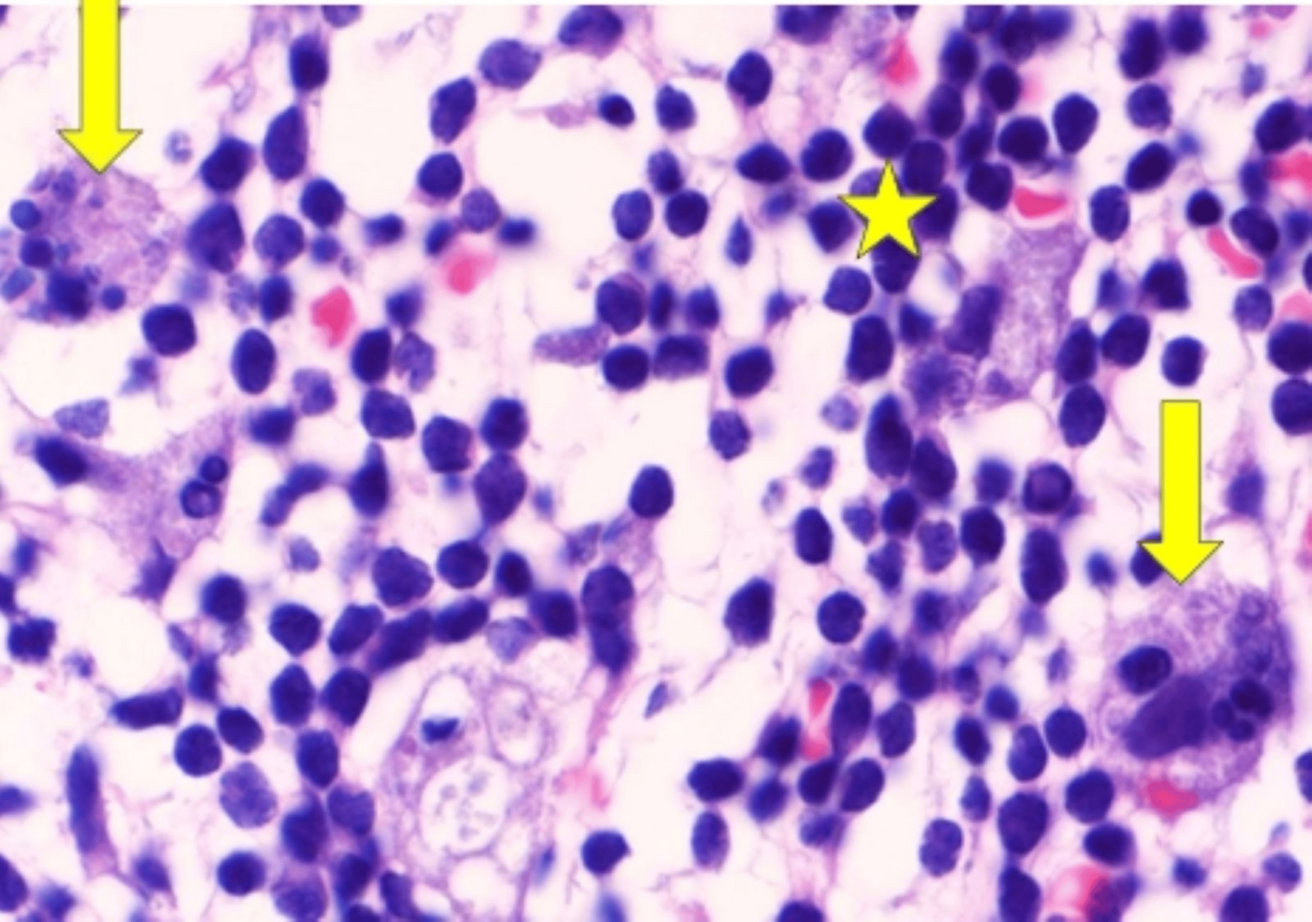
Bone marrow biopsy showing marrow extensively filled with leukemic cells (star) and hemophagocytosis (arrow) using Wright-Giemsa stain with 1000x magnification.

Flow cytometric analysis demonstrated an abnormal population of clonal B lymphocytes (clonal cluster of differentiation (CD)5/CD23-positive B-cell population), with lambda light chain restriction. Lambda light chain restriction suggests predominance of B cells that produce lambda light chains compared to kappa light chains, ultimately suggesting monoclonal cell proliferation. Bone marrow biopsy demonstrated a high burden of active CLL, with 92% of abnormal clonal B lymphocytes and prominent hemophagocytosis (Figure 2), suggestive of HLH.

In addition, the patient also met other clinical criteria for HLH, including hyperpyrexia, splenomegaly, bicytopenia, hypertriglyceridemia with hypofibrinogenemia, hemophagocytosis in bone marrow, ferritin >500, and soluble CD25 (IL-2 receptor) >2400 U/mL. Appropriate anti-mold, fungal, bacterial, and opportunistic infection treatment was initiated. Due to active infections and pancytopenia, CLL and HLH treatment was limited. The patient was initially treated with rituximab, intrathecal methotrexate, intravenous immunoglobulin, and intravenous dexamethasone and later started on ibrutinib (Imbruvica), a Bruton’s tyrosine kinase inhibitor, and venetoclax, a B-cell lymphoma 2 (BCL-2) protein inhibitor, to further control CLL progression upon discharge. At the 16-month follow-up visit, the patient remains alive without any recurrence of HLH disease. Labs remained stable for CLL as well during the follow-up period.

## Discussion

Etiologies of HLH can involve genetic predisposition or acquired forms associated with a wide variety of infections, autoimmune disorders, or malignancy. Multiple cases of infections solely associated with HLH are reported in the literature, including fungal and viral infections [2,8], e.g., HLH in a patient with influenza [9], tuberculosis [10], aspergillosis [11], Epstein-Barr virus (EBV) [12], cytomegalovirus (CMV) [13], human immunodeficiency virus (HIV) [14], and *Candida* [15]. A case of infection associated with HLH in patients with hematological malignancies was also found in the literature, such as a case of EBV-associated HLH and development of invasive aspergillosis and death in a patient with hematological malignancies in X-linked lymphoproliferative disease [16]. In a retrospective observational study of 71 HLH patients admitted to the intensive care unit, 18 (25%) patients developed invasive aspergillosis, which was more correlated with HLH with infection cases (n = 8) rather than lymphoma-associated HLH and HLH of unknown origin [17]. Cases of HLH in CLL have also been reported. It can occur if the disease has been left untreated [18,19] or the disease is in progression stage [4,20], or it can happen with the ongoing infection. Additionally, it can happen during the CLL treatment phase (ibrutinib [21,22], fludarabine or rituximab [23]), relapse, or seen in a refractory case, or it can be identified during the autopsy. CLL patients are immunodeficient and commonly have IgG deficiency, prone to infections [24]. Our patient had a triad of multiple infections in the setting of IgM deficiency, HLH disease, and CLL progression.

In our case, the disease course was somewhat confounding, as the patient appeared to have progression of CLL, but also presented with multiple superimposed infections. Our patient presented with a white cell count of 35,000, which progressively increased to 93,000, and gradually developed pancytopenia during the single hospitalization, suggestive of CLL progression leading to the development of HLH. The patient initially presented with renal failure requiring hemodialysis, and had complete recovery of renal function with dialysis. His tumor lysis syndrome resolved as well. The patient was started on antifungals and antivirals. The dilemma at first was to diagnose the patient with HLH in the setting of multiple infections, given that some diagnostic parameters can be reactive. The patient met seven out of eight criteria for the diagnosis based on HLH protocol: temperature ≥38.5°C; splenomegaly; cytopenias affecting at least two of three lineages in the peripheral blood; hemoglobin <9 g/dL; platelets <100 × 10^9/L; neutrophils <1.0 × 10^9/L; fasting triglycerides >265 mg/dL and/or fibrinogen ≤1.5 g/L; hemophagocytosis in bone marrow, spleen, liver, lymph nodes, or other tissues; low or absent natural killer (NK)-cell activity; serum ferritin concentration ≥500 μg/L; and elevated soluble CD25 (soluble IL-2 receptor) ≥2400 U/mL [25]. These were mainly the classification criteria developed using the best available knowledge for clinical trial enrollment for familial HLH and may not be reliable for malignancy-associated HLH (M-HLH) diagnosis, have unknown sensitivity and specificity, and require a validation study [26]. High serum ferritin should raise a suspicion for this disease, especially when >500 ug/L [27]. Early recognition is imperative, and follow-up testing, such as soluble IL-2 receptor, should be ordered to help cinch the diagnosis. In the event the patient had a systemic fungal infection, additional laboratory testing may be helpful. There was a case of pulmonary aspergillosis in the literature leading to acute respiratory distress syndrome in an immunocompromised patient using steroids and having HLH, which was identified using serum galactomannan. They insisted on including various techniques to identify fungal infections, such as direct examination, culture, polymerase chain reaction (PCR), and antigen detection, as the sensitivity of tests varies [28]. A study conducted at the Mayo Clinic on CLL patients found that patients who were also diagnosed with invasive aspergillosis have a median survival of less than one year [29]. Therefore, it is highly recommended to rule out fungal ideology, with a thorough workup for viral diseases, along with investigation for bacterial infections in a patient suspected of having HLH disease.

The strategy was to both address the CLL progression as well as treat the concurrent infections to address all possible underlying causes. Since HLH and malignancy have overlapping features, diagnosis needs clinical judgement to proceed with early testing if suspected [27]. Providing trigger-specific treatment on time, as well as early immunosuppressive medications and immunochemotherapy to reduce the massive inflammatory response, can drastically improve survival. Patients with HLH associated with infections may also benefit from immunoglobulin therapy [30,31]. HLH-2004 guidelines recommend initial chemo-immunotherapy with etoposide, dexamethasone, and cyclosporine, and in selected patients with intrathecal methotrexate and corticosteroids. Regardless, the HLH-94 protocol (based on pediatric protocols) has also been applied in the clinical setting, which consists of a combination treatment of dexamethasone, etoposide ± cyclosporine, and in some cases rituximab (anti-CD20 antibody). The treatment regimen itself comes with a lot of possible complications, including worsening cytopenia, increasing infection risk/precipitating worsening cytokine production, and cyclosporine in the setting of renal failure would not be recommended. Ideally, it is better to ensure the patient is improving from an infectious standpoint first, prior to initiating chemo/immunotherapy. In our case, given the tenuous clinical status, rituximab, intravenous immunoglobulin (IVIG), and intrathecal methotrexate were given in the inpatient setting, followed by the addition of Bruton’s tyrosine kinase inhibitor (BTKi) in the outpatient setting to address CLL.

Newer biological agents targeting hyperinflammatory pathway suppression, which may be beneficial on an individual basis, are available but need randomized clinical trials to prove further benefits, e.g., anakinra (interleukin-1 receptor antagonist) [32], ruxolitinib (Janus-associated kinases 1 or 2 inhibitor) [33], tocilizumab (a monoclonal IL-6 receptor antibody) [34], alemtuzumab (anti-CD52 monoclonal antibody) [35], infliximab (immunosuppressant) [36], etanercept (immunosuppressant) [37], baricitinib (Janus kinase JAK1/ JAK2 inhibitor) [38], and emapalumab (monoclonal antibody against interferon-γ) [39]. These approaches must be individualized to the patient at hand, taking into account performance status and toxicity profile. Our approach was more so treating the underlying causes with reservation of HLH-directed treatments (such as etoposide), reserved for the refractory setting. Untreated cases have high mortality within a year, while with treatment, survival can be improved.

## Conclusions

Secondary HLH can present with various symptoms and should be suspected in patients with hematological malignancy when presenting with cytopenia, failure to thrive, and organ failure. This case is unique as the patient's critical illness was initiated by unprotected exposure to mold, fungus, and other organisms while renovating a 200-year-old house. The CLL progression also ties in with multiple active infections, which certainly speaks to the crucial role of education in patients with malignancy-induced immunodeficiency disorders.
